# Supplementary material for: Molecular evolution across developmental time reveals rapid divergence in early embryogenesis
Source: Evol Lett. 2019 Jun 19;3(4):359–73. doi: 10.1002/evl3.122 (PMC6675142; doi:10.1002/evl3.122)
Supplement: Supplementary file 1 — Supplementary Table S1. List of ModENCODE C. elegans transcriptome datasets used in analysis of differential expression across development. Supplementary Figure S1. Distributions of polynomial parameter values from expression profile function fits for each gene (α = overall expression level, β1 = linear change over time, β2 = quadratic curvature, β3 = cubic S‐shape to expression profile over development). Supplementary Figure S2. Biplots of per‐gene polynomial fit parameter values, colored by coexpression module (as in Fig. 4 main text), show clustering of genes with similar parameter values (α = overall expression level, β1 = linear change over time, β2 = quadratic curvature, β3 = cubic S‐shape to expression profile over development). Supplementary Figure S3. Enrichment of early embryonic expression categories defined by Baugh et al. (2003) among coexpression modules. Note that not all categories are mutually exclusive. Supplementary Figure S4. Cumulative distribution of non‐synonymous site substitutions (log‐transformed K A) for each coexpression module illustrates the distinct incidence of extremely low K A values for M4 and M6 (top panel), indicating the subset of genes with little protein sequence divergence between C. elegans and C. briggsae. Supplementary Figure S5. Rates of protein evolution (K A, log‐scale) plotted as a function of the polynomial fit parameter values to the expression time series (α = overall expression level, β1 = linear change over time, β2 = quadratic curvature, β3 = cubic S‐shape to expression profile over development). Supplementary Figure S6. Transcriptome divergence index (TDI and TDI*) shows lowest values at timepoints 7 (180 min) and at timepoint 1. The adult stage (timepoint 30) shows one of the highest values. [file EVL3-3-359-s001.docx]

# Supplementary Information

**Supplementary Table S1**. List of ModENCODE *C. elegans* transcriptome datasets used in analysis of differential expression across development.

| ModENCODE ID | Dataset | Stage or embryo age treatment (h) |
| --- | --- | --- |
| 4527 | L1 (4h post-L1) | L1 |
| 4574 | L1 (4h postL1) | L1 |
| 4530 | L2 (17.75h post-L1) | L2 |
| 4575 | L2 (17.75h post-L1) | L2 |
| 4532 | L3 (26.75h post-L1) | L3 |
| 4576 | L3 (26.75h post-L1) | L3 |
| 4534 | L4 (34.25h post-L1) | L4 |
| 4535 | L4 (34.25h post-L1) | L4 |
| 4581 | Young adult (pre-gravid, 46h post-L1) | adult |
| 4593 | Young adult (pre-gravid, 46h post-L1) | adult |
| 6561 | N2_EE_50-0 | 0 |
| 6568 | N2_EE_50-30 | 30 |
| 6579 | N2_EE_50-60 | 60 |
| 6585 | N2_EE_50-90 | 90 |
| 6562 | N2_EE_50-120 | 120 |
| 6563 | N2_EE_50-150 | 150 |
| 6564 | N2_EE_50-180 | 180 |
| 6565 | N2_EE_50-210 | 210 |
| 6566 | N2_EE_50-240 | 240 |
| 6567 | N2_EE_50-270 | 270 |
| 6569 | N2_EE_50-300 | 300 |
| 6570 | N2_EE_50-330 | 330 |
| 6571 | N2_EE_50-360 | 360 |
| 6572 | N2_EE_50-390 | 390 |
| 6573 | N2_EE_50-420 | 420 |
| 6574 | N2_EE_50-450 | 450 |
| 6575 | N2_EE_50-480 | 480 |
| 6576 | N2_EE_50-510 | 510 |
| 6577 | N2_EE_50-540 | 540 |
| 6578 | N2_EE_50-570 | 570 |
| 6580 | N2_EE_50-600 | 600 |
| 6581 | N2_EE_50-630 | 630 |
| 6582 | N2_EE_50-660 | 660 |
| 6583 | N2_EE_50-690 | 690 |
| 6584 | N2_EE_50-720 | 720 |

**Supplementary Table S2**. Summary of gene ontology (GO) term enrichment for each co-expression module.

**Supplementary Table S3**. Summary of phenotype enrichment analysis (PEA) terms for each co-expression module.


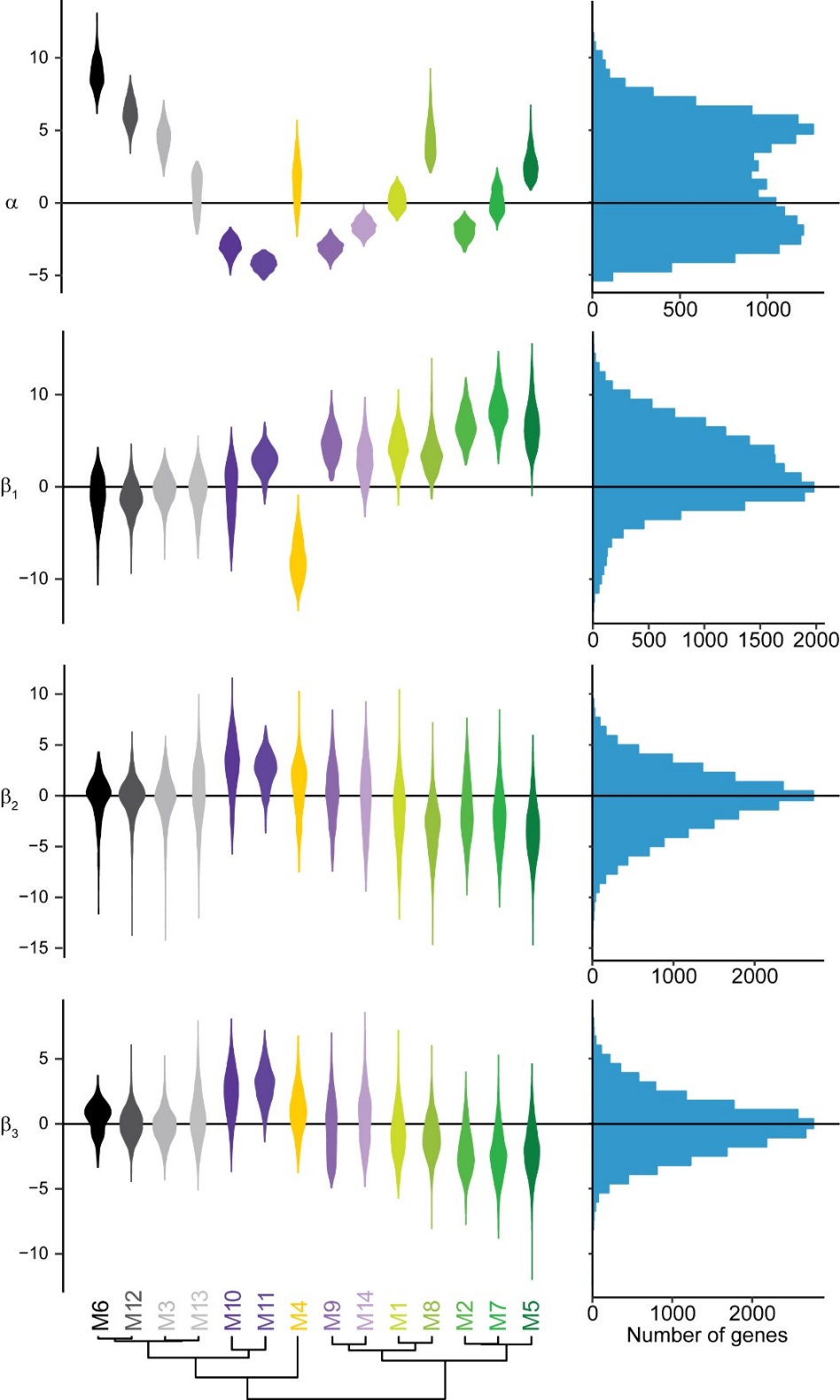


**Figure S1**. Distributions of polynomial parameter values from expression profile function fits for each gene (α = overall expression level, β_1_ = linear change over time, β_2_ = quadratic curvature, β_3_ = cubic S-shape to expression profile over development). Violin plots show the parameter value densities separately for each co-expression module (see Figure 1 in main text).

**
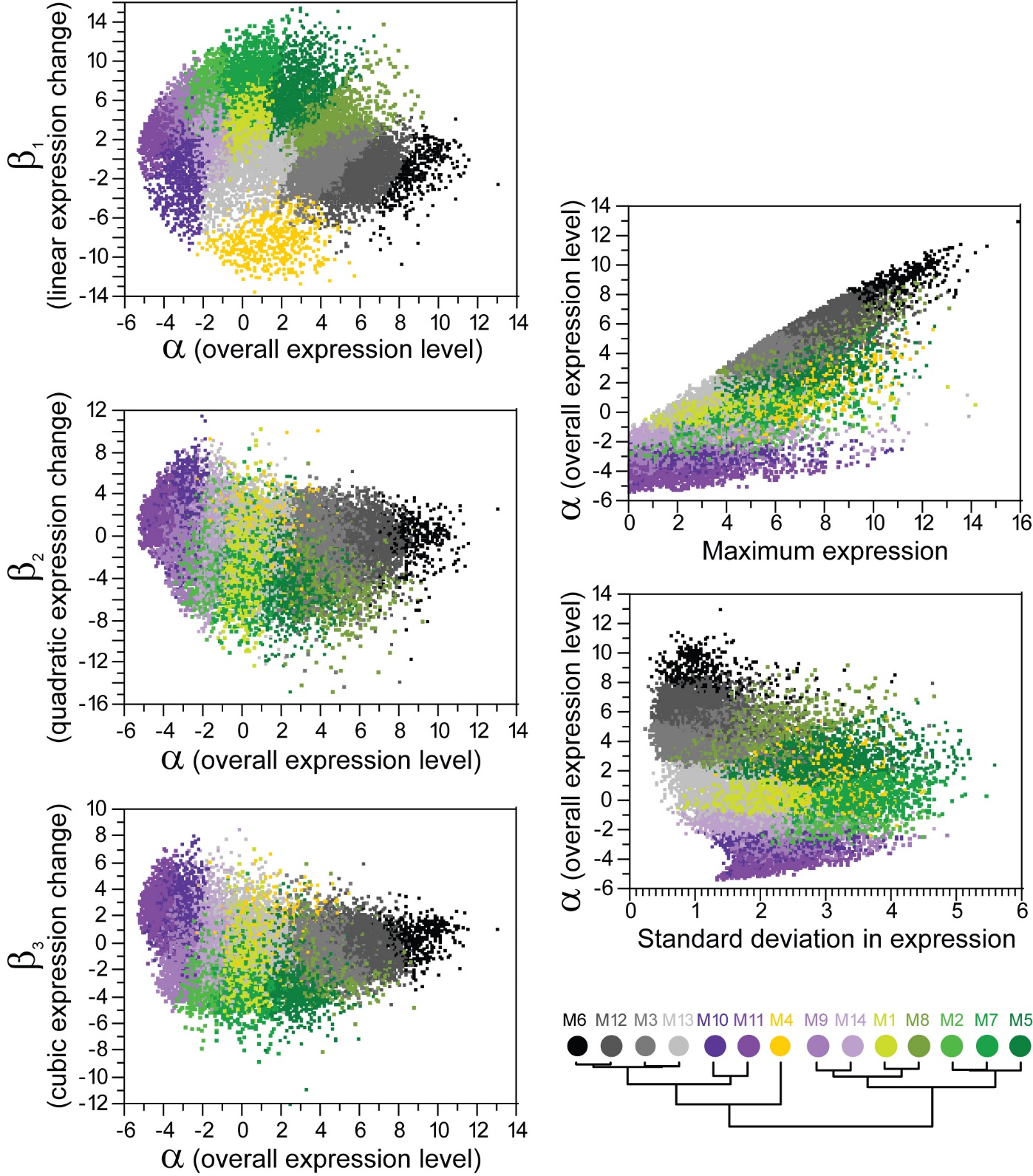

Supplementary Figure S2**. Biplots of per-gene polynomial fit parameter values, colored by co-expression module (as in Figure 4 main text), show clustering of genes with similar parameter values (α = overall expression level, β_1_ = linear change over time, β_2_ = quadratic curvature, β_3_ = cubic S-shape to expression profile over development). The α parameter correlates positively with a measure of maximum expression level across timepoints and has a complex relationship with variation among timepoints in expression level.


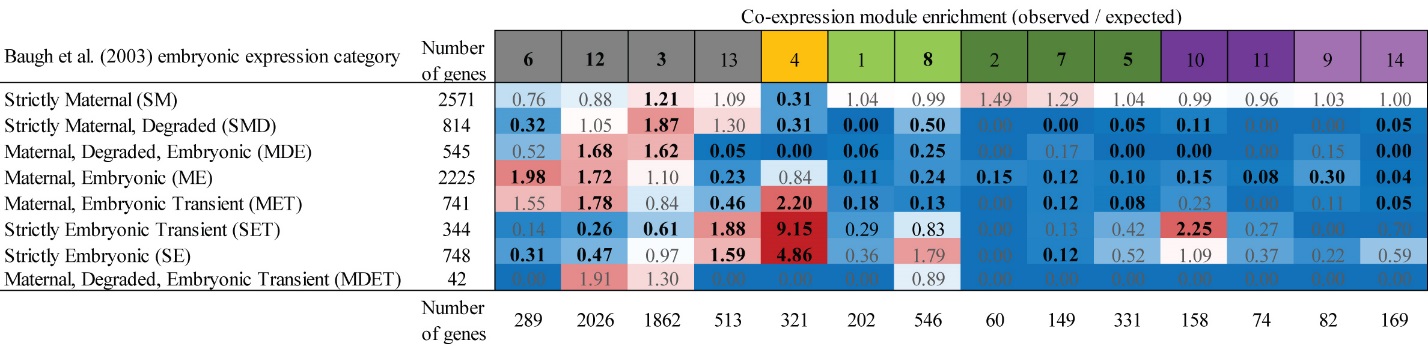


**Supplementary Figure S3**. Enrichment of early embryonic expression categories defined by Baugh et al. (2003) among co-expression modules. Note that not all categories are mutually exclusive. Values in bold black text significant, gray text values non-significant (χ^2^-test, df=1, Bonferroni-corrected α=0.00052). Module number colored as in Figure 1 of the main text.


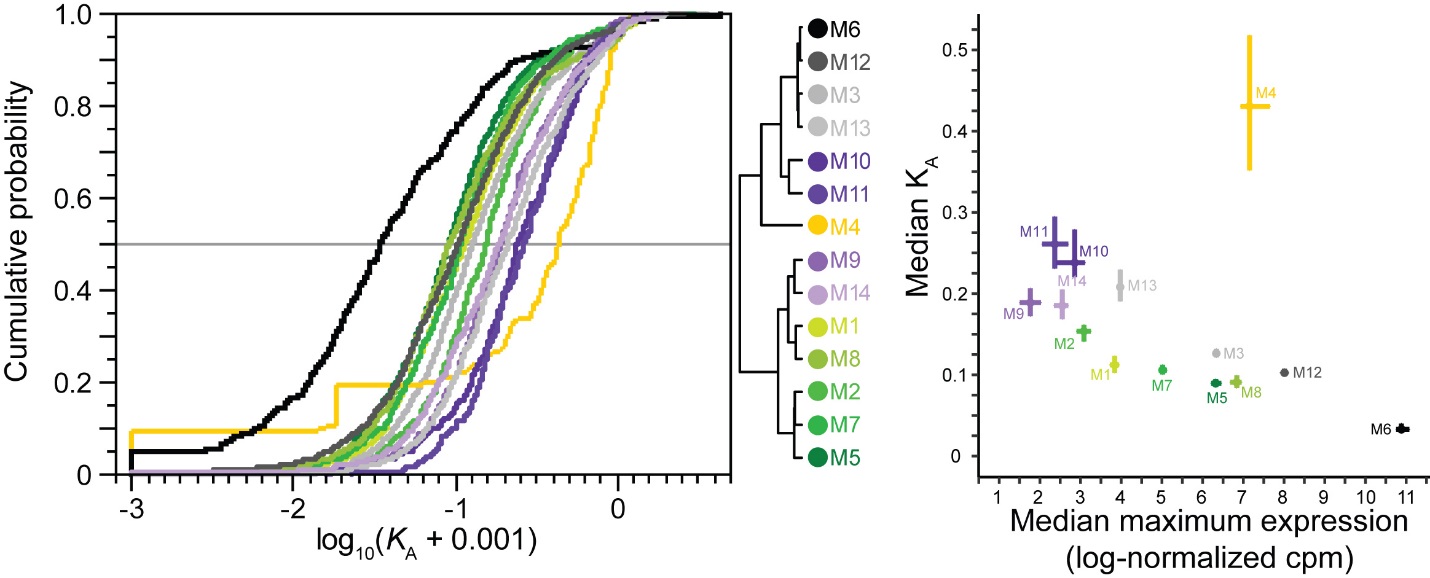


**Supplementary Figure S4**. Cumulative distribution of non-synonymous site substitutions (log-transformed *K*_A_) for each co-expression module illustrates the distinct incidence of extremely low *K*_A_ values for M4 and M6 (top panel), indicating the subset of genes with little protein sequence divergence between *C. elegans* and *C. briggsae*. Otherwise, genes in M6 have a low median rate of evolution whereas genes in M4 have a high median rate of evolution (horizontal line indicates 50^th^ percentile). Modules containing genes with higher average expression tend to have slower rates of evolution (bottom panel); note M4 as outlier to this trend. Verbal models suggest that genes will be more likely to diverge in coding sequence when they have fewer *cis*-regulatory elements, and when coding mutations are less likely to exert pleiotropic effects (Carroll 2008). The flat expression profiles of genes in the constitutive modules that we identified (M6, M12, M3, M13) versus the dynamic profiles of the modules that get up- or down-regulated across ontogeny implies the potential for simple versus complex *cis*-regulatory control. Indeed, for a given magnitude of expression, genes in modules with simple profiles tend to show faster coding sequence evolution (e.g. M3 vs M5, M13 vs M1; see also Figure 4B), though genes with simple profiles often have high expression and slow evolution (e.g. M6).


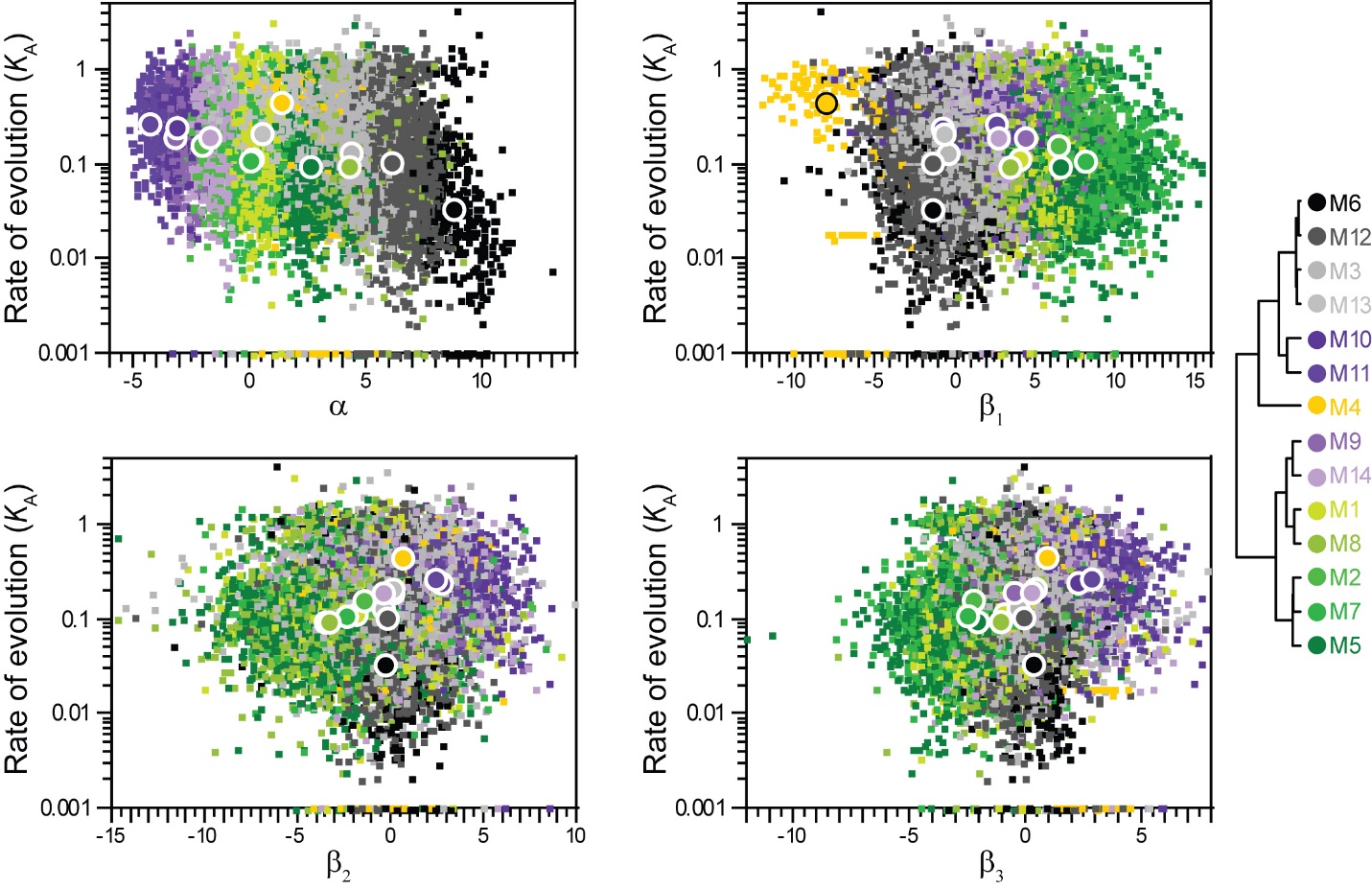


**Supplementary Figure S5**. Rates of protein evolution (*K*_A_, log-scale) plotted as a function of the polynomial fit parameter values to the expression time series (α = overall expression level, β_1_ = linear change over time, β_2_ = quadratic curvature, β_3_ = cubic S-shape to expression profile over development). Per-gene values shown as small squares, module median values shown as large circles. Dendrogram indicates expression similarity among co-expression modules.


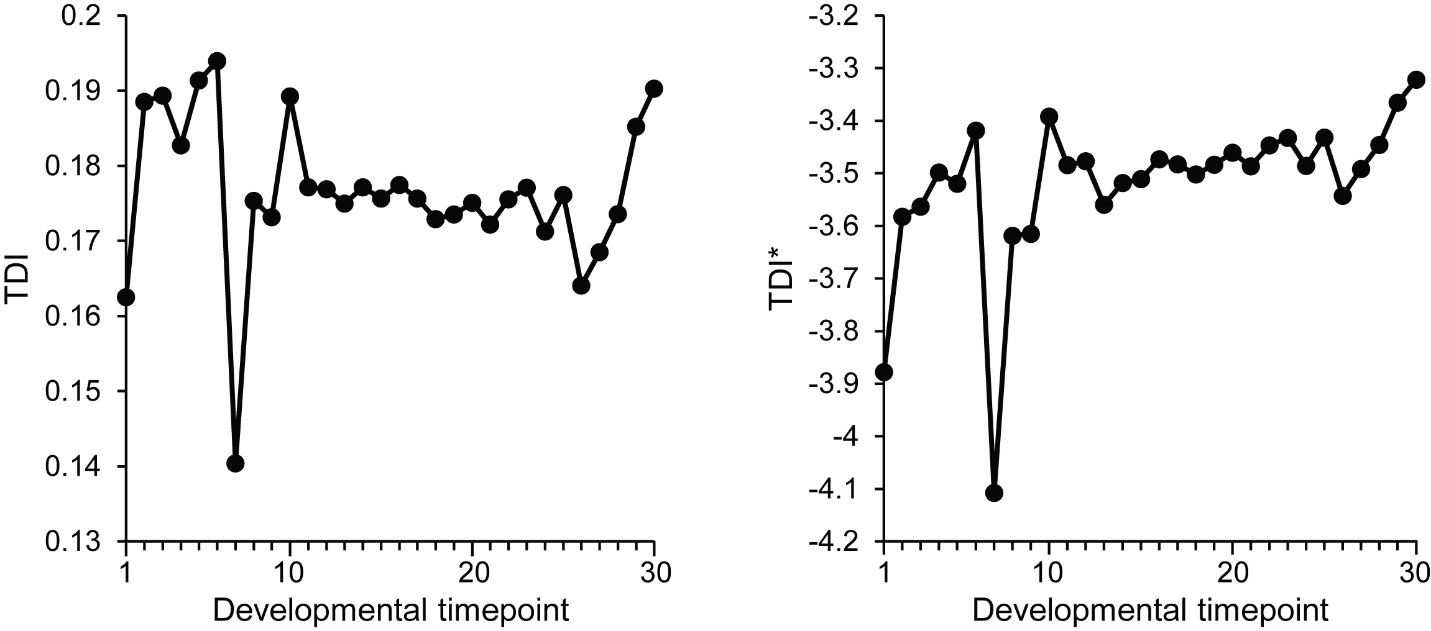


**Supplementary Figure S6**. Transcriptome divergence index (TDI and TDI*) shows lowest values at timepoints 7 (180 min) and at timepoint 1. The adult stage (timepoint 30) shows one of the highest values. The TDI is the expression-weighted average coding sequence divergence (*K*_A_ for 12,628 orthologs between *C. elegans* and *C. briggsae*) for a given timepoint *t*, calculated as ${TDI}_{t}=\frac{\sum_{i=1}^{n} \left[ K_{Ai}\cdot E_{it} \right]}{\sum_{i=1}^{n} \left[ E_{it} \right]}$ and ${TDI}_{t}^{*}=\frac{\sum_{i=1}^{n} \left[ {log}_{2}\left( K_{Ai}+0.001 \right)\cdot E_{it} \right]}{\sum_{i=1}^{n} \left[ E_{it} \right]}$, based on all *n*=12,628 genes with gene expression of gene *i* at time *t* (*E*_it_) quantified on a log_2_ scale.
